# Effect of *Satureja montana* Essential Oil on Model Lipid Membranes

**DOI:** 10.3390/biom15010005

**Published:** 2024-12-24

**Authors:** Simona Sennato, Silvia Trabalzini, Maria Gioia Fabiano, Domenico Truzzolillo, Edouard Chauveau, Cecilia Bombelli, Federica Rinaldi, Maria Carafa

**Affiliations:** 1 CNR-Institute for Complex Systems, Piazzale Aldo Moro 5, 00185 Rome, Italy; 2 Department of Physics, Sapienza University of Rome, Piazzale Aldo Moro 5, 00185 Rome, Italy; 3 Drug Chemistry and Technology Department, Sapienza University of Rome, Piazzale Aldo Moro 2, 00185 Rome, Italy; sil.trabalzini@gmail.com (S.T.); mariagioia.fabiano@uniroma1.it (M.G.F.); maria.carafa@uniroma1.it (M.C.); 4 Laboratoire Charles Coulomb, UMR 5221, CNRS–Université de Montpellier, 34095 Montpellier, France; domenico.truzzolillo@umontpellier.fr (D.T.); edouard.chauveau@umontpellier.fr (E.C.); 5 CNR-Institute for Biological Systems, Secondary Office of Rome-Reaction Mechanisms, c/o Chemistry Department, Sapienza University of Rome, Piazzale Aldo Moro 5, 00185 Rome, Italy; cecilia.bombelli@cnr.it

**Keywords:** *Satureja montana* essential oil, lipid monolayers, Langmuir isotherm, lipid-oil interaction

## Abstract

*Satureja montana* essential oil is a natural substance able to inhibit the growth of several pathogens. This antimicrobial effect is often attributed to its ability to penetrate cellular structures and disrupt them. Although these properties are recognized as playing a key role in the mechanism of action of this substance, many unresolved issues still exist, and fundamental studies focused on such aspects are scarce. In this framework, we investigated the interaction of SEO with lipid monolayers, which represent simplified models of cell membranes, using the Langmuir monolayer technique, complemented by fluorescence anisotropy and differential scanning calorimetry on lipid bilayers. By focusing on packing conditions that approximate those of biological membranes and using lipids with different polar heads and structures, such as the ones occurring in bacterial membranes, we aim to clarify the effect of this essential oil on the lipid membrane. Our results show that *Satureja montana* essential oil consistently manages to insert into the membrane and interfere with the lipid–lipid interactions, thereby altering the lipid packing and significantly increasing the membrane fluidity, depending on the oil concentration and the nature of the lipid.

## 1. Introduction

Natural substances extracted from plants have proven to be excellent sources of bioactive compounds with antioxidant, antimicrobial, anti-tumour and anti-inflammatory properties [1]. Their abundant availability, combined with a minimal environmental burden, makes them a valid, efficient, and ecologically safer alternative to many synthetic products. Furthermore, many essential oils have demonstrated broad-spectrum antimicrobial potential by targeting key factors of pathogenicity and drug resistance. Consequently, there is widespread agreement that essential oils could provide an effective solution for combating antimicrobial resistance. The latest reports confirm their strong direct-killing or re-sensitizing potential, offering a means to replace or rejuvenate the otherwise fading arsenal of antibiotics [2], so these substances are increasingly viewed as novel weapons against microbial growth and biofilm formation [3,4,5,6].

It is worth noting that many essential oils have already been included in the treatment of various disorders and infections, serving as permeation enhancers for topical and transdermal delivery systems in combination with drugs. Essential oils offer the advantage of reduced or negligible toxicity compared to chemical penetration enhancers (see [7] and references therein). Here, the efficacy of delivery depends on the ability to overcome the barrier functions of the skin. Therefore, structural modifications to the skin membrane induced by oil are crucial for enabling drug delivery [8].

Several mechanisms of permeation enhancement have been hypothesised, including lipid disruption in the stratum corneum by the constituents of essential oils and the formation of complexes between the enhancer and the drug, or structures in the stratum corneum. It is widely accepted that the penetration-enhancing activity of the oil is concentration-dependent, a property that supports its use as both an antimicrobial and therapeutic adjuvant.

*Satureja montana* essential oil (SEO) is extracted from plants belonging to the genus *Satureja*, mainly located in the eastern part of the Mediterranean area. Oils obtained from different species of the genus *Satureja* express different degrees of biological properties related to their metabolite composition [9]. Different biological activities have also been observed depending on the extraction method, as they influence the effective composition of the final oil mixture [10]. In general, the volatile fraction of SEO is mainly composed of phenolic compounds, e.g., thymol and carvacrol, which are part of the natural defence system against several pathogens in plants and are associated with the bioactive properties of the oil [11]. Thus, the effective antimicrobial activity of SEO against plants and foodborne pathogens results from the high content of active phytochemicals [12].

It is well known that the high hydrophobicity of SEO, like that of most essential oils, enables its accumulation in cell membranes. This property is considered directly related to the mechanism of action of essential oils. Regarding SEO, there is strong evidence of its inhibition of the growth of several pathogens, as reported in various articles [9,13,14,15], but only a few of them have addressed its mode of action. A recent study demonstrated that the antimicrobial activity of SEO is associated with rapid and reversible damage to the cell membrane of *S. aureus*, which compromises the integrity of the membrane, highlighting this as a key mechanism of action [16].

Some plant extracts, such as SEO, show higher antimicrobial activity with respect to pure compounds, which is probably due to the peculiar synergy occurring in multi-component mixtures [17]. The powerful antimicrobial activity of plant extracts has been attributed to the richness of carvacrol and its isomer thymol [18,19]. Many investigations on pure carvacrol report its significant ability to disturb membrane integrity and permeability; this leads to the leakage of cellular components, changes in fatty acids and phospholipids composition, impairment of energy metabolism, and influences on the synthesis of genetic material [20]. Moreover, a loss of membrane potential due to an exchange between protons and potassium has been observed [18]. This effect has been directly correlated with the presence of the hydroxyl groups in carvacrol and thymol, which act as transmembrane monovalent cation carriers.

The ability of essential oils to integrate into cellular structures and disrupt the membrane is pivotal in determining their antimicrobial effectiveness. Therefore, analysing the effect of SEO on membranes is a fundamental step in addressing many unresolved issues related to its biological activity. Currently, studies focused on this aspect are still scarce. Many challenges associated with the interaction of membranes with natural compounds can be addressed by using model membrane systems known as Langmuir monolayers [21]. These monomolecular films, formed at the air–water interface, are widely used because they simplify the complexity of the cell membrane. They also enable the easy manipulation of chemical composition and surface density and allow for the investigation of the influence of bioactive molecules on membrane properties under controlled experimental conditions [22].

In the present study, we used a commercial SEO sample, which demonstrated significant activity against *Escherichia coli, Staphylococcus aureus, and Listeria monocytogenes* bacteria and biofilms. This activity was evident through dramatic morphological changes, such as sunken, malformed, or collapsed cells, along with damage to the cell surface or the formation of pores [14]. As the first step to shedding light on the basic aspects of the interaction between SEO and the bacterial cell membrane, we prepared Langmuir monolayers at the air–water interface by using lipids with different charges and structures, such as dimyristoyl-*sn*-glycero-phosphocholine (DMPC), dimyristoyl-*sn*-glycero-phosphoethanolamine (DMPE), dimyristoyl-*sn*-phosphatidylglycerol (DMPG), and cardiolipin. Choline-based lipids, used in combination with PE, PG, or phosphatidylserine (PS) and cardiolipin, are often used to model bacteria membranes [23,24]. Among them, PG and cardiolipin are typical of the membranes of *S. aureus* and *S. pneumoniae* [25]. This investigation was complemented by differential scanning calorimetry and fluorescence anisotropy measurements performed on lipid bilayers. The scope of this work is to understand how SEO affects the thermodynamic properties of a monolayer, such as its molecular packing, fluidity, and stability, depending on the nature of the lipid polar head, under the highly simplified condition of a single-component monolayer. This represents an initial, yet fundamental, study of this commercial sample of SEO. In future works, complex monolayer systems such as mixtures of saturated and unsaturated PC, PE, PG and CL, will be investigated to develop more realistic models of the membrane of bacteria.

## 2. Materials and Methods

### 2.1. Chemicals

**Figure 1:**
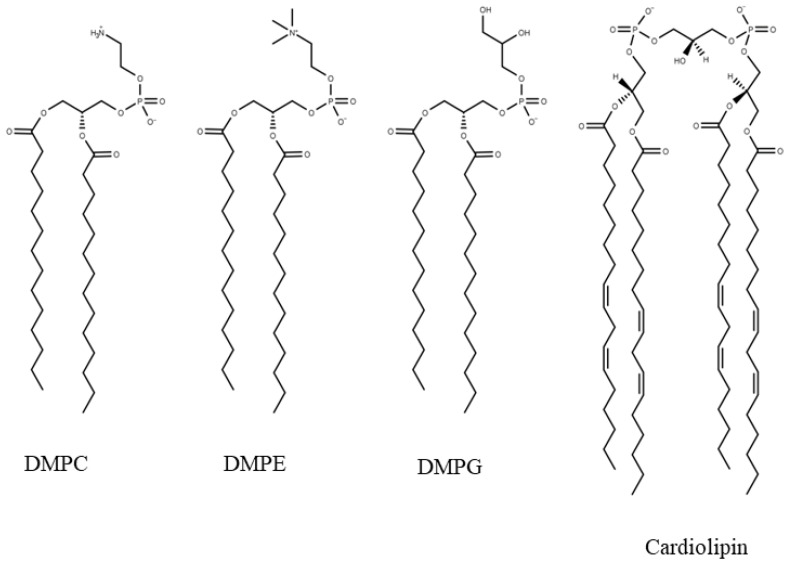
Chemical structure of the lipids. The structure of cardiolipin refers to the most abundant species.

Chemical structures of the lipids employed in this investigation are shown in Figure 1. Moreover, 1,2-dimyristoyl-*sn*-glycero-phosphocholin (DMPC), 1,2-dimyristoyl-*sn*-glycero-3-phosphoethanolamine (DMPE), 1,3-bis(sn-3’-phosphatidyl)-*sn*-glycerol, and cardiolipin (CL) were purchased from Sigma-Aldrich (Milan, Italy); 1,2-dimyristoyl-*sn*-glycero-3-phosphorylglycerol sodium salt (DMPG-Na) was a kind gift from Lipoid (Germany). The melting temperatures of DMPC, DMPE, and DMPG were determined as the temperatures of the peak maxima, measured by differential scanning calorimetry (as described in Section 2.4), and corresponding to 24 °C, 50 °C, and ≃19 °C, respectively. The CL lipid was extracted from a natural source (bovine heart), with a TLC purity >97%, and fatty acid purity >80%. The most abundant chains were characterised by two unsaturated bonds in positions 9 and 12 (18:2 c9,12). This justifies a melting temperature out of the range used in calorimetry and lower than the other lipids employed in this study [26], as already observed in previous investigations [27,28].

All the lipids were used without further purification. DMPC and CL were dissolved in chloroform, DMPE, and DMPG in a mixture of chloroform/methanol (4:1 volume/volume), at a concentration of 1 mg/mL. Research-grade chloroform, ethanol, and methanol were supplied by Sigma-Aldrich (Milan, Italy) and were used as received.

In all experiments, the subphase was 0.01 M HEPES, prepared using Milli-Q grade deionized water (Millipore) at a pH of 7.40 ± 0.05. At this condition, both DMPC and DMPE can be assumed neutral.

*Satureja montana* essential oil (SEO) was purchased from a commercial source (Talia, Roma, Italy, http://www.taliaessenze.com). The composition of this sample is quite complex, including oxygenated monoterpenes such as carvacrol (43.9%), thymol (7.6%), thymol methyl ether (4.3%), borneol (3.1%), and significant amounts of monoterpene hydrocarbons (24.2%) [14]. SEO was dissolved in ethanol at a concentration of 33% *v*/*v* (stock solution) and then diluted in ethanol at the desired final concentrations for the experiments (from 0.03 to 0.6% *v*/*v*, corresponding to 0.31–5.6 mg/mL).

### 2.2. Monolayer Studies

Langmuir monolayers were prepared using a KSV Minitrough (KSV Instrument, Helsinki, Finland) placed on an anti-vibration table and enclosed in a Plexiglass box to reduce surface contamination. Compression was achieved via the symmetric movements of two opposing Delrin barriers at a constant rate of 50 mm/min. The surface tension of the lipid monolayer, commonly named surface pressure Π, was measured by using the Wilhelmy method, using a filter paper plate, with an accuracy of 0.1 mN/m. The trough and the barriers were thoroughly cleaned before each measurement with appropriate solvents and abundantly rinsed with ultra-pure water. Before lipid deposition, the surface was cleaned by slowly sweeping the barriers and vacuum aspirating the surface in between, until no change in surface pressure was detectable when comparing the closed and open positions. All the experiments were performed at fixed temperatures (20 ± 0.5 °C) by using a water bath circulator. No precaution to prevent CL oxidation was taken. The results reported here represent the averages of three different isotherms, at least. For all the lipids, we ascertained that the overall reproducibility of the isotherms was around 1–2 mN/m, with the largest variability observed for the highest concentration of added SEO.

The monolayers were prepared at the air–water interface following the Langmuir technique [29]. An appropriate amount of the lipid solution (around 20 μL) was spread by a micro-syringe on the aqueous subphase. For lipid monolayers in the absence of SEO, the solvent was allowed to evaporate for about 5 min before starting the compression.

Preliminary experiments were performed to understand the best experimental conditions. The deposition at the interface of a mixed SEO–lipid solution in organic solvent showed a scarce reproducibility of the isotherm, due to the high volatility of SEO at the interface and consequent loss of material, as observed for other essential oils [30]. For this reason, we preferred the injection of SEO in the water subphase under a preformed lipid monolayer. We also verified the perturbation to surface pressure induced by the injection of different solvents capable of diluting SEO. To this aim, 50 μL of pure ethanol, methanol, or chloroform were injected under the subphase. Since ethanol alone did not cause a significant pressure perturbation, SEO was diluted in this solvent. The experimental protocol consisted of the SEO injection under the monolayer at Π≃0 of a fixed volume of SEO, at the desired concentration, and the measure of the isotherm after stabilisation of the surface pressure.

#### Analysis of Compressibility

The change of the surface tension with the area of the surface associated with a liquid film can be attributed to the interfacial elasticity [31]. For a Langmuir monolayer, elasticity is related to the compressibility of the condensed monolayer phase. Analogously to the bulk compressibility, the compressibility of monolayers can be defined as follows:

(1) $$C_{s}=-\frac{1}{A_{m}}{\left(\frac{\partial A_{m}}{\partial \Pi }\right)}_{T}$$

where $A_{m}$ is the molecular area. The reciprocal quantity of $C_{s}$, denoted as the surface compression modulus *K*, i.e.,

(2) $$K=C_{s}^{-1}=-A_{m}{\left(\frac{\partial \Pi }{\partial A_{m}}\right)}_{T}$$

can be determined in a simple way from the slope of the Π-$A_{m}$ isotherms. *K* is often used to characterise the monolayer properties as it measures the ability of the monolayer to store mechanical energy as stress. The compressibility is different as the monolayer changes phases, one can see that K→0 (or $C_{s}\to \infty$) at the LE–LC transition, while at the LC–S phase transition, *K* changes discontinuously [31]. *K* was calculated from the isotherm data by taking numerical derivatives of the Π-$A_{m}$ isotherms using the Differentiate tool in Origin 8.6 software. The numerical derivatives were smoothed with a Savitzky–Golay filter with a five-point window.

### 2.3. Penetration Experiments

We investigated the effect of SEO penetration into lipid films through relaxation experiments, examining the behaviour of the surface pressure over time at a constant molecular area in pure lipid films and after the injection of SEO. Analysing the relaxation of the pure lipid monolayers also helps to reveal occurrences of lipid desorption or instability [32,33]. Lipid monolayers were compressed up to a certain value of Π, fixed as a target, and then 20 μL of ethanolic solution of SEO was added soon after reaching this value. Different concentrations in the range of 0.03 to 0.6% were tested. The observed variations in surface pressure over time can be correlated with SEO penetration at the interface, as the injection of ethanol alone does not induce any change.

The penetration of SEO in the film was evaluated by considering the surface pressure increment, defined as $\Delta \Pi =\Pi_{SEO}-\Pi_{0}$, where $\Pi_{SEO}$ and $\Pi_{0}$ are the values reached in the presence or absence of SEO, after film stabilization.

### 2.4. Fluorescence Anisotropy and Differential Scanning Calorimetry on Lipid Bilayer Membranes

For further evaluation of the interaction of SEO with lipid membranes, we used lipid bilayer vesicles (liposomes) as model membrane systems. This allowed us to study the variation in fluidity by measuring the fluorescence anisotropy of the diphenylhexatriene (DPH) probe and assess the thermotropic behaviour using Differential Scanning Calorimetry (DSC).

Fluorescence anisotropy of DPH was measured using an LS55 spectrofluorometer (PerkinElmer, MA, USA) at $\lambda_{exc}$ = 380 nm and $\lambda_{em}$ = 425 nm, at 20 °C, and calculated according to the following equation:

(3) $$A_{F}=\frac{I_{vv}-GI_{vh}}{I_{vv}+2GI_{vh}},$$

with $I_{vv}$ and $I_{vh}$ denoting the intensities of the emitted fluorescence (arbitrary units), parallel and perpendicular to the direction of the vertically polarised excitation light, respectively, and $G=I_{hv}/I_{hh}$ being a correction factor, to be determined experimentally before each measurement as the ratio between the vertically and horizontally polarised emission components, for a given horizontally polarised incident beam. The fluorescence anisotropy values $A_{F}$ are inversely proportional to the membrane fluidity; lower values correspond to low structural orders and/or lower membrane viscosity [34]. Prior to the experiments, we confirmed that the addition of SEO at the working concentrations did not induce changes in total fluorescence intensities.

Lipid bilayer membranes were formed by dissolving lipids in a chloroform/methanol solution at a concentration of 10 mg/mL. An appropriate amount of DPH prepared at 2 mM was added to lipids to obtain a lipid:probe ratio of 1000:1. According to a protocol described in a previous study [35], after removing the organic solvent, the lipid film was rehydrated in HEPES buffer at pH 7.4 and then extruded to obtain unilamellar bilayer vesicles. SEO (5 mg/mL) was added after vesicle formation to obtain approximately a 1:2 lipid:SEO molar ratio, then the sample was kept in the dark under constant low stirring at 20 °C for one hour to promote the interaction with the bilayer, before performing anisotropy measurements. The liposome sizes were evaluated using dynamic light scattering measurements (see Appendix A.3). For each sample, the anisotropy value represents the average over 30 measurements taken during a total acquisition time of 300 s.

DSC experiments were performed in the same conditions, with the only difference being the use of multilamellar bilayers, which have been shown to exhibit the same thermotropic behaviour as unilamellar vesicles but with a higher signal, as proven in past studies [35]. To investigate the SEO–lipid interaction, we added SEO at a 5 mg/mL concentration to the suspension of lipid vesicles, prepared at a concentration of 10 mg/mL. As an additional test, we measured the thermograms of SEO–lipid mixed membranes, where the oil was added directly during lipid film preparation by dissolving it in the organic solvent at the same mass ratio.

DSC measurements were carried out using a TA Q2000 calorimeter by performing three heating/cooling cycles under nitrogen flux via a modulated temperature protocol (amplitude = 0.3 °C, rate = 2 °C/min), over a 60 s period in the temperature range 10–70 °C. Excess molar heat capacity was calculated after baseline adjustment and normalization to the total lipid concentration. The melting transition temperatures ($T_{m}$) of the different samples were determined at the peak maxima of the heat capacity curves.

## 3. Results

### 3.1. Pressure–Area Isotherms

Preliminarily, we verified that under the experimental conditions used in our study, SEO exhibits negligible surface activity. The control is motivated by the nature of the essential oil used in the study, which is a commercial sample composed of several compounds, with carvacrol being the most abundant (43%). Carvacrol is an oxygenated monoterpene with high partition coefficients of 3.64 and 3.26, determined in octanol–water and liposome–buffer phases, respectively, showing a strong preference for hydrophobic phases [18]. The measured surface pressure of SEO spread on the air–water interface reached lower values at the end of compression (see Appendix A.1) compared to the surface pressure isotherm of the lipid monolayer, indicating that the surface activity is primarily due to the presence of lipid monolayer at the interface.

**Figure 2:**
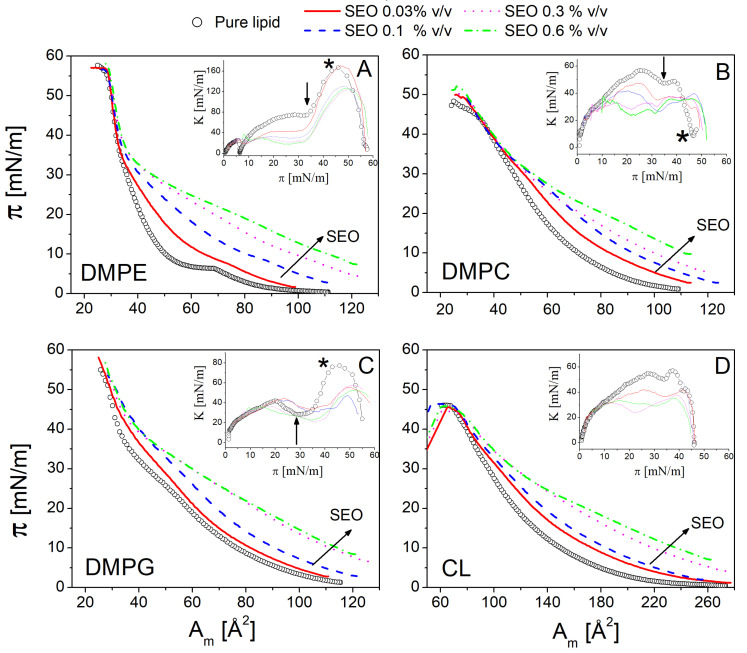
Surface pressure–area Π-$A_{m}$ isotherms and surface compression modulus *K* (inset) recorded for DMPE (**A**), DMPC (**B**), DMPG (**C**), and CL (**D**) monolayers, in the absence of SEO (open symbols), and at different concentrations of SEO injected at null surface pressure (0.03—red, 0.1—blu, 0.3—pink and 0.6—green % *v*/*v*; see legend and colour online). The arrow in the main panel marks the increasing concentration of SEO. In the insets, the vertical arrows and the asterisks highlight the LE-LC and LC-solid transitions, respectively (see text).

The surface pressure–molecular area (Π-$A_{m}$) isotherms of DMPE, DMPC, DMPG, and CL on a HEPES subphase are shown in Figure 2 for the different amounts of SEO injected. As a general observation, in all the lipid monolayers, the addition of SEO constantly causes a shift of the curves toward a larger molecular area, or equivalently, an increase in surface pressure at a fixed molecular area. This is the most evident proof of the inclusion of SEO in the monolayer. First, SEO causes a regular increase in the lift-off area of the isotherm, indicating that a larger number of molecules are present at the interface. This demonstrates that SEO interacts with the lipids and that its insertion promotes the expansion of the monolayers, with a more pronounced effect observed at higher concentrations.

The isotherms of the different lipids exhibit distinct characteristics, depending on the nature of the lipids. Figure 2A shows the isotherms of the zwitterionic DMPE—pure and with SEO. The curve of pure DMPE contains all the typical features of a Langmuir isotherm. Starting from a large area (or low pressure), the monolayer behaves as a two-dimensional gas, where lipid molecules are far apart and do not interact with each other, resulting in almost negligible surface pressure. Then, the compression causes the onset of the liquid phase, where the variation in the slope of the curve marks the liquid expanded (LE) to the liquid condensed (LC) phase transition, with lipid molecules becoming more ordered and tightly packed. Upon further compression, the film reaches the solid state, characterised by a steep slope, until it collapses at a molecular area close to 28 Å^2^ (at a surface pressure Π≃ 57 mN/m). In the presence of the lowest SEO concentration (0.03% *v*/*v*), the LE-LC transition becomes almost undetectable and shifts to a larger molecular area. In the solid state, beyond a surface pressure of approximately 45 mN/m, all the curves overlap, suggesting the expulsion of SEO from the interface without film rupture, i.e., the structure of the film is maintained. It is interesting to note that, in the solid state, SEO cannot be retained in the film at any of the explored concentrations, indicating that the penetration capability of SEO strongly depends on the lipid packing state.

To further elucidate the film behaviour, the surface compressional modulus K of the monolayers was calculated according to Equation (2) (inset of Figure 2A). This parameter provides complementary information on the variation of the thermodynamic and structural properties of the monolayer. As expected, for a pure DMPE monolayer, this parameter becomes vanishingly small at the LE-LC transition, at a surface pressure close to 7 mN/m. Then it progressively increases until an abrupt variation occurs in correspondence with the LC-S transition (see the arrow in the inset of Figure 2A), reaching its maximum value where the collapse emerges, before declining again. In the presence of SEO, the values of K decrease regularly in all thermodynamic phases of the monolayer, as a consequence of the weakening of lipid packing caused by SEO, evidencing a “fluidifying” effect. The overall fluidization of the monolayer is further reflected in the continuous decrease of the absolute maximum of K with increasing SEO content [31,36]. A similar decrease in values of K for lipid monolayers has been reported in the literature for other essential oils and drug–lipid mixed films [37].

In the isotherm, the kink at 7 mN/m, which marks the LE-LC transition of DMPE, disappears even with the smallest addition of SEO. Similarly, the maximum value of K (see the asterisk in the inset of Figure 2A), which indicates the monolayer collapse, shifts to higher surface pressure values at an SEO concentration larger than 0.03% *v*/*v*. This testifies to the concentration-dependent disordering effect of SEO. A closer look at the behaviour of compressional modulus reveals that the effect of SEO depends on the packing state of the monolayer. In the LE phase, at a surface pressure lower than 15 mN/m, the variation of K is rather independent of the SEO amount, as a large number of molecules can fit into a loosely packed monolayer without significantly altering the packing. With increasing surface pressure in the range of 15–35 mN/m, corresponding to the more interesting LC phase, the response becomes concentration-dependent. Notably, a three-fold reduction in K is observed at a surface pressure of around 30 mN/m, which is commonly considered equivalent to the packing of biological membranes [38,39]. At a surface pressure greater than 35 mN/m (solid phase), no variation is observed between the different curves at higher concentrations, further supporting the expulsion of SEO from the film beyond 0.03% *v*/*v*. It is interesting to note that SEO expulsion is accompanied by a reduction in K, suggesting that the interaction between the expelled SEO and the lipid interface persists. This is likely due to the possible localisation of SEO at the outer region of hydrophobic chains, which may weaken the attraction between hydrocarbon chains, thereby reducing the mechanical resistance of the film. This effect occurs only when a sufficiently large amount of SEO is added since the modification of film surface organisation in a very closely packed condition requires a sufficiently large perturbation to overcome the high mechanical resistance of the film. Overall, the addition of SEO to the subphase alters the thermodynamic and structural properties of the DMPE monolayer by modifying phase behaviour, molecular packing effectiveness, and membrane fluidity. A similar effect was also observed with DPPE in the presence of other natural compounds, such as geraniol [37].

The isotherm of the zwitterionic lipid DMPC is shown in Figure 2B. The surface pressure increases regularly with the molecular area, as observed for DMPE. The isotherm does not show any phase transition but remains in its LE state as it approaches the solid state, as reported in previous investigations at different temperatures [40]. These studies indicate the presence of the LE-LC phase transition below 12.5 °C with its progressive disappearing from 15.8 °C to 20 °C. After SEO injection, the isotherm shifts to the larger area and changes its shape with a decrease in the values of K (see inset of Figure 2B), which is particularly evident in the surface pressure range of 15–30 mN/m. These effects demonstrate the modification of lipid–lipid interactions, the onset of lipid–SEO associations, and the fluidizing effect of SEO on the film. In the more compact state, the isotherm remains unchanged, but a slight effect on the surface compression modulus still occurs. This further supports the idea that, despite its expulsion, SEO stays near the lipid interface and influences the film by weakening the intermolecular interactions, thereby facilitating film ordering and compaction at lower molecular packing, as observed for DMPE. Finally, it can be noted that the local minimum of K (see the arrow in the inset of Figure 2B), which marks the transition to a solid state for the DMPC monolayer, shifts to a lower surface pressure or larger molecular areas with increasing SEO concentration.

The behaviour of the anionic lipid DMPG is shown in Figure 2C. The isotherm of the pure lipid shows a barely noticeable signature of the LE-LC transition beyond 20 mN/m, which corresponds to the decrease in K after the first local maximum, observed at around 35 mN/m (see the arrow in the inset Figure 2C). Then, a noticeable increase in the slope of the isotherm marks the transition to the solid state and the rapid increase of K (see the asterisk in the inset of Figure 2C). The effect of SEO addition is less regular compared to the other two lipids. At the two lower SEO concentrations, 0.03 and 0.1%, the isotherms shift to a larger molecular area, with minimal variation in the slope, especially in the LE region. The variation in slope and the shift of the curve become more pronounced at 0.3 and 0.6% of SEO, where a maximum is reached. At 0.03 and 0.1% *v*/*v*, K shows a shift to a higher surface pressure at the first local maximum, which marks the barely visible LE-LC transition. This indicates that the low amount of SEO, in a loosely packed state, finds accommodation which increases resistance to compression and stiffens the film, likely because it reduces the repulsive interaction between the polar heads. As surface pressure increases, closer packing is enforced, causing the loss of this optimal SEO accommodation, likely leading to SEO penetration at the level of hydrocarbon chains and a decrease in the compression modulus, i.e., an increase in film fluidity. It is interesting to note that the absolute maximum of each curve shifts to higher pressures, thus providing further evidence of the disturbance in lipid-lipid interactions caused by SEO insertion, which impedes the film’s transition to a more compact state. As the amount of SEO increases, a reduction in the value of K is observed at all surface pressures, indicating an overall disturbing effect on the packing of the film.

The isotherm of the anionic lipid CL is similar to the one of DMPG but it shifted at larger molecular areas due to the unique structure of this Gemini lipid (Figure 2C). As observed previously, the effect of SEO addition is regular and concentration-dependent, with a progressive disturbance in molecular packing, evident in the expansion of the monolayer and the reduction of the surface compression modulus.

**Figure 3:**
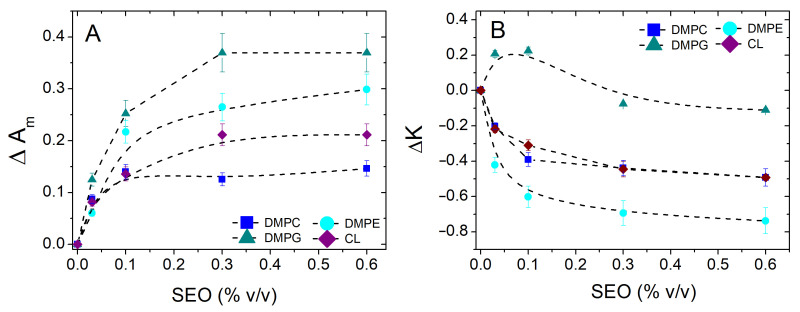
Relative variation in the molecular area $\Delta A_{m}$ (panel **A**) and surface compression modulus K (panel **B**) calculated at surface pressure Π= 30 mN/m for different SEO concentrations, for DMPC (*■*), DMPE (•), DMPG (*▲*), and the CL lipid (*♦*).

To provide an overview and compare the effect of SEO on the different monolayers, for all the isotherms shown in Figure 2, we extrapolated the molecular area at Π = 30 mN/m, i.e., the surface pressure mimicking the cell membrane packing, and calculated its relative variation as $\Delta A_{m}=\left(A_{m}^{SEO}-A_{m}^{0}\right)/A_{m}^{0}$, where $A_{m}^{0}$ is the area in the absence of SEO. By a similar definition, we also calculated the relative variation in surface compression modulus K at the same target pressure. The results are shown in Figure 3, panels A and B, respectively. The positive increment of $\Delta A_{m}$ provides immediate evidence of the presence of SEO at the interface, indicating its insertion into the film and perturbation of molecular packing, regardless of the nature of the lipid. A concentration-dependent effect, with distinct features for each lipid, is always observed. The largest effect is observed for DMPG. Saturation occurs above 0.3% *v*/*v* for most lipids, except for DMPC, where it appears even at lower concentrations (0.1% *v*/*v*). In general, the presence of saturation can be intuitively explained by considering that the perturbation caused by the injection of a certain number of molecules can create voids and vacancies, which can then accommodate a larger amount of SEO, so the perturbation does not increase further. Another possibility is that at higher SEO concentrations, the localization of SEO molecules shifts towards the region of hydrophobic carbon chains, to which SEO has a higher affinity, thereby reducing the effect of interfacial disturbance. This hypothesis is consistent with the regular behaviour of isotherms with increasing amounts of SEO and with the observed superimposition of the curves at higher SEO concentrations.

Figure 3B shows the relative variation of the surface compression modulus at 30 mN/m. This parameter helps to shed light on the effect of SEO on the mechanical response of the film in the condensed phase. Here, positive values of ΔK correspond to film tightening, while negative values indicate film fluidization. It is immediately evident that the greatest fluidizing effect is found for DMPE and the smallest for DMPG. While a regular reduction is observed for all lipids, DMPG exhibits non-monotonic behaviour with concentration. DMPC and CL show an intermediate fluidizing effect, likely connected to the different molecular reorganizations of these molecules. That SEO incorporation can induce such varied effects depending on the lipid’s nature is intriguing. The presence of a complex set of interactions between SEO and lipids can generate either a local loosening of the film or compaction, depending on the nature of the polar head. Interestingly, a tightening effect is observed at a low SEO concentration in DMPG. At these concentrations, SEO expands the film while increasing its rigidity. Up to a certain threshold, the SEO-DMPG interaction enhances film rigidity, likely due to strong electrostatic correlations that promote a more ordered structure. Beyond this threshold, excess SEO creates a disturbance, resulting in a slight fluidizing effect, similar to that observed in other lipids.

### 3.2. Relaxation Experiments and Penetration of SEO into Monolayers

The injection of SEO into monolayers always results in a strong increase in surface pressure due to its rapid penetration into the film, followed by a prolonged relaxation until a stable value is reached (see Appendix A.2). We compare the effect of the SEO injection at two different conditions, at Π∼ 0 mN/m, where the almost null lipid–lipid interactions should favour the maximal penetration of SEO, and at Π = 30 mN/m, where the oil interacts with a lipid monolayer in the packing condition that mimics those of biological cell membranes.

The observed behaviour is complex and often not regular. This may be attributed to the unique properties of the essential oil used, which is a multi-component mixture of various molecules and can produce unusual effects when interacting with lipids. Furthermore, in pure lipid films at Π = 30 mN/m, a large decrease in the surface pressure is observed. This is not surprising because the instability of the film increases at high surface pressure due to lipid dissolution or diffusion in the subphase [32,33].

**Figure 4:**
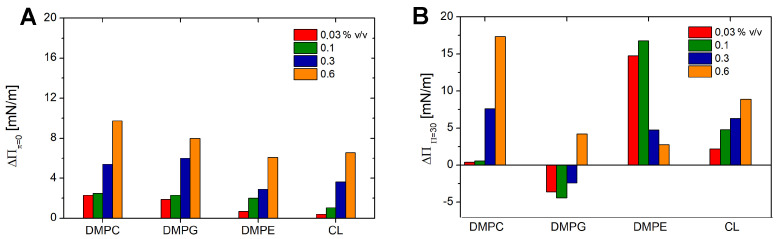
Increment in the surface pressure after injection of SEO under monolayers at surface pressures Π= 0 mN/m (**A**) and Π= 30 mN/m (**B**), for all the investigated lipids at the SEO concentrations, as indicated in the legend.

To evidence the effect of SEO injection and take into account the relaxation toward equilibrium of the pure lipid monolayers, we calculated the surface pressure increment ΔΠ, as defined in Section 2.3. The results are shown in Figure 4. As expected, SEO injection at Π= 0 mN/m induces a positive variation in the surface pressure in a concentration-dependent behaviour. Within the tested concentration range, saturation does not occur, suggesting that the film can accommodate an even higher number of molecules. The small differences observed among the different lipids are likely due to the weak SEO–lipid coupling at the surface pressure.

Significant differences and irregular behaviour occur at higher surface pressures (Figure 4B). DMPG shows a negative increment because the final pressure of the mixed SEO–lipid film is lower than that of the pure lipid film. This effect has to be related to the ”disappearance” of molecules from the interface due to lipid loss or connected to the formation of SEO–lipid complexes expelled from the film. It may be hypothesised that the hydrophobic attraction between SEO and hydrocarbon chains pulls lipid molecules out of the film, thereby reducing the number of molecules at the interface and, consequently, the surface pressure. Beyond a certain oil concentration, a positive increment is observed again. This suggests that the density increases because oil penetration resumes. This may indicate that, following partial lipid depletion, the film becomes capable of accommodating additional SEO molecules.

DMPC and CL exhibit a nearly consistent behaviour, with a progressively increasing positive increment in surface pressure as SEO concentration increases. For DMPE, the pressure increment peaks at intermediate SEO concentrations and then decreases. This may result from the disappearance of SEO from the interface at high concentrations, as the closely packed film can accommodate only a limited number of molecules, causing any excess to escape from the interface.

## 4. Fluorescence Anisotropy and Differential Scanning Calorimetry on Lipid Bilayers

The DPH probe inserts in the hydrophobic core, parallel to the acyl chains of phospholipids or in the centre of the membranes, at 7.8 Å from the centre of the bilayer [41,42]. Thus, variations in DPH anisotropy can be linked to structural changes in the hydrophobic core of the bilayer. For this reason, this probe is commonly used in model membranes to estimate the degree of bilayer order and fluidity.

**Figure 5:**
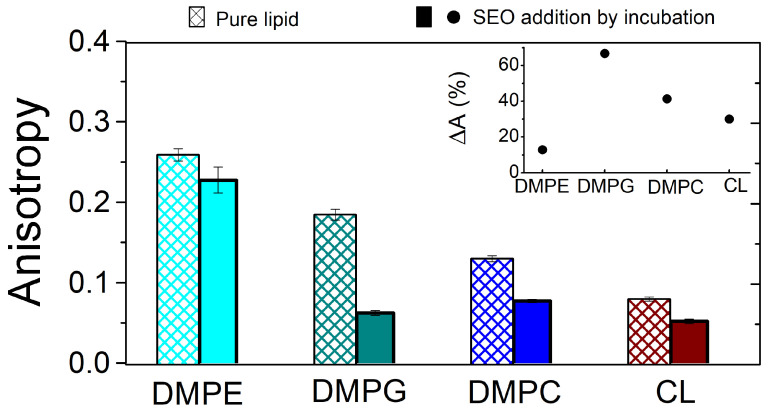
Values of fluorescence anisotropy at 20 °C for DMPE, DMPG, DMPG, and CL lipid membranes in the absence of (striped bars) and after (full bars) interaction with SEO, with SEO incubated in the vesicle suspensions. The inset shows the percentage change of anisotropy ΔA.

Figure 5 displays the fluorescence anisotropy of DPH in pure lipid membranes and after SEO interaction. The inset shows the percentage change of anisotropy, calculated as $\Delta A=\left(A_{0}-A_{SEO}\right)/A_{0}\cdot 100$, where $A_{0}$ and $A_{SEO}$ are the values obtained before and after the addition of SEO, respectively. After 1 hour of incubation in lipid vesicle suspensions, SEO reduces the DPH anisotropy in all the bilayers, indicating a fluidizing effect at the level of the hydrocarbon chains. The extent of the reduction depends on the specific lipid, with the largest effect observed for DMPG, followed by DMPC, DMPE, and CL, which show similar variations.

The large effect observed in DMPG indicates that SEO penetrates to a large extent in the hydrophobic region, probably favoured by the large lipid fluctuations due to the proximity to the melting transition ($T_{m}∼19\;{}^{\circ}$C; see Section 2.1).

For the other lipids, a minor variation is observed. In DMPE, the gel state of the bilayer ($T_{m}=50\;{}^{\circ}$C), in connection with a certain degree of ordering of the zwitterionic heads, prevents the deep penetration of SEO, which probably remains adsorbed at the level of the polar heads, favoured by attractive interactions with the PE dipole. A similar situation could be hypothesised for DMPC. For this lipid, the proximity to the transition temperature ($T_{m}=24\;{}^{\circ}$C) should favour SEO insertion, as observed for DMPG. On the other hand, the reduction is lower and it could be connected to the occurrence of cation–π interactions between SEO and the choline polar head, thereby favouring the localization of the oil close to the polar head and limiting its deep penetration in the hydrophobic region [43]. Lastly, the liquid state and high fluidity of the hydrocarbon chains in CL can explain the minimal variation in anisotropy observed after the addition of SEO. When the percentage change of anisotropy is calculated for each lipid, the difference between DMPG and the other lipids is even more striking. This quantity can be assumed to be an index of the degree of interaction between the SEO and the hydrophobic portion of the lipid bilayer.

**Figure A3:**
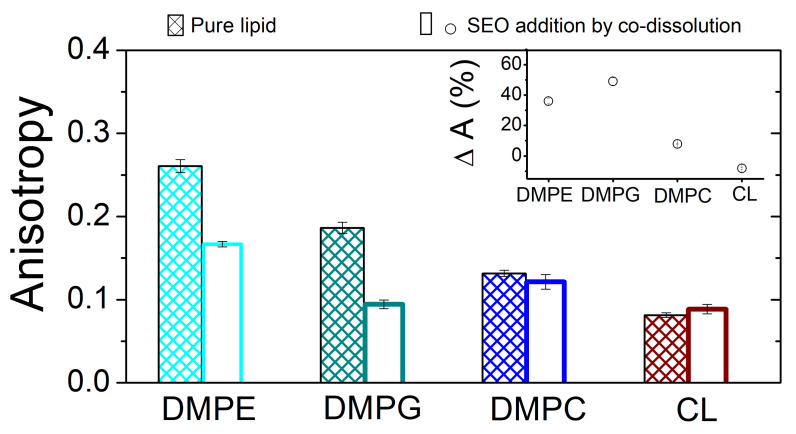
Values of fluorescence anisotropy at 20 °C for DMPE, DMPG, DMPG, and CL lipid membranes in the absence (striped bars) and after (full bars) interaction with SEO, with SEO dissolved in the organic solvent with the lipid, in the initial step (film formation) of vesicle preparation. The inset shows the percentage change of anisotropy ΔA.

Similar results were found with a different experimental protocol for adding SEO, which involves co-dissolving SEO and lipids in the organic solvent during the initial step of vesicle preparation (see Appendix A, Figure A3). This condition allows for the maximum miscibility between the oil and lipids due to the minimization of the SEO–lipid interaction.

**Figure 6:**
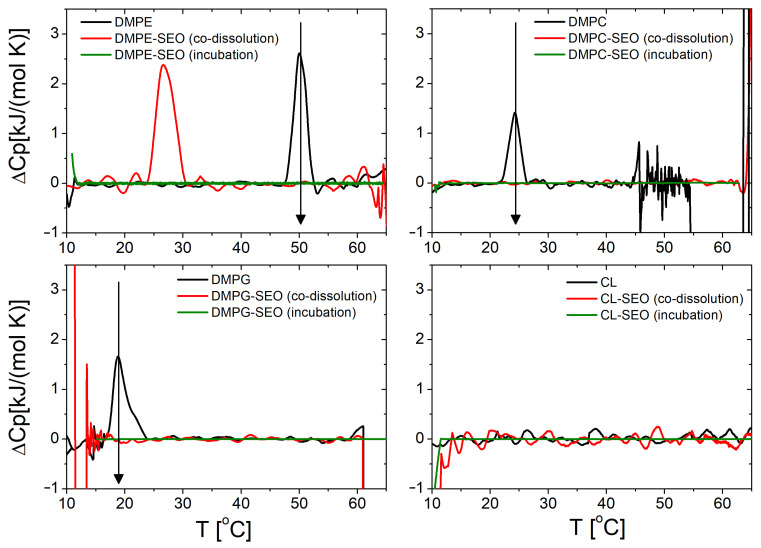
Excess molar heat capacity $\Delta C_{p}$ for pure DMPE, DMPC, DMPG, and CL lipid membranes, and in the presence of SEO, added during membrane formation by co-dissolution in the organic solvent and on already formed vesicles, by incubation in the vesicle suspension (see legend). Vertical arrows mark the lipid transition temperature.

DSC measurements demonstrate a significant impact on the thermotropic behaviour of lipid vesicles under the conditions investigated. Firstly, we note that our calorimetric protocol did not detect any transition for CL lipids, which likely occurs outside the probed temperature range. Excluding CL, the addition of SEO by incubation on already-formed lipid vesicles eliminates the DSC transition peak (indicated by the vertical arrow in Figure 6). This finding indicates the loss of any form of cooperativity between lipids, probably due to the large amount of added molecules. As part of further testing, DSC measurements were also performed by adding SEO during vesicle formation through the co-dissolution of SEO and lipids in the organic solvent. Under this condition, only for DMPE is it observed that the transition shifts to lower temperatures, from ≃50 to ≃26 °C, but it is still observable, while for the other lipids, no transition is observed. These results corroborate the large fluidizing effect of SEO on the bilayers and open the way for a deeper calorimetric investigation that examines different SEO concentrations and protocols of interaction, to obtain thermodynamic information that could be useful in clarifying the effects of this molecule on the different lipids.

## 5. Discussion

The chemical structure and the proportion of the components of a natural compound determine its ability to perturb cell membranes [44]. Interactions between the components in a mixture can determine synergistic, antagonistic, or additive effects, thereby altering the antimicrobial activity of the substance. For this reason, a mixture or a total extract of a natural compound can exert a different antimicrobial effect than its particular components. These characteristics complicate the understanding of the details of the mechanism of action of essential oils. Our findings provide evidence of strong interaction and modification of lipid packing by SEO across all the investigated lipids. The insertion of the oil is facilitated by the favourable hydrophobic attraction between the aliphatic chains of the lipids and its primary components, mainly carvacrol, thymol, and monoterpene hydrocarbons [14]. Some differences are observed in dependence on the nature of the polar head of the lipid, which modulates the complex set of interactions. To obtain insight into these differences at a molecular level, one may refer to the few existing studies on the main components, i.e., carvacrol and thymol. It has been reported that the interaction of carvacrol and thymol with lipids, and their consequent effects on membrane stability and antimicrobial activity, are driven by the presence of a hydroxyl group conjugated to an aromatic ring (a system of delocalised electrons), which, in turn, determines their ability to release proton [18]. Calculations via density functional theory (DFT) indicated that in thymol and carvacrol, the presence of this group confers a more negative delocalized charge than the positive delocalized charge [45]. This feature is common to other polyphenols with aromatic rings, such as resveratrol, which possesses a wide spectrum of antimicrobial activity and can decrease the membrane surface charge upon insertion [46]. The presence of the hydroxyl group is responsible for the cell wall breakdown that characterizes the antibacterial action of carvacrol [18]. This group also affects the cytoplasmic membrane’s chemical and physical properties, as well as the bilayer’s stability and the proton passive flux through the cell membrane [18].

All the lipid monolayers undergo SEO insertion, leading to a significant expansion after SEO addition, which decreases molecular ordering. This finding clearly supports the occurrence of an effective interaction between SEO and lipid monolayers, even in a close-packed film. As observed, Brewster angle microscopy observations of zwitterionic DPPC and anionic DPPS monolayers after thymol addition indicate that the expansion is also associated with the formation of lipid aggregates. These aggregates may represent the seeds for structural destabilization of the membrane and loss of its biological functions [24].

The greatest effect in terms of packing disturbance is observed in DMPG, where the largest variation in both molecular area and anisotropy, along with a distinct behaviour of surface pressure upon insertion, is found. The large effect can be correlated to the peculiar SEO-DMPG interaction, influenced by the anionic charge of this lipid, particularly when considering the weakly acidic character of SEO. It has been reported that the $pK_{a}$ of carvacrol is in the range of 9–10, meaning that at a neutral pH, only about 0.1% of carvacrol molecules are dissociated [37]. Albeit minimal, the onset of electrostatic repulsion between the anionic DMPG polar head and SEO could favour the formation of a regular arrangement and promote an overall film expansion. Furthermore, at 20 °C, near the melting temperature, the liquid state of the monolayer likely facilitated the penetration of the oil and subsequent film modification. Further support for the role of electrostatic repulsion among polar heads can be found in surface-specific vibrational spectroscopy investigations on thymol and anionic monolayers composed of DPPS, such as the study conducted by Ferreira and coworkers [24]. This study reports on a more prominent effect of the SEO interaction in the phosphate region of the spectra, where the electric charge is localised, compared to other regions of the phospholipid. These considerations highlight the need for further investigations into the effect of temperature on the interaction between monolayers and this essential oil, which has not been explored yet.

In the ordered gel-like DMPE monolayer, a minor effect is observed. The lower interfacial film elasticity of DMPE can motivate the larger resistance to the insertion of SEO, which modulates the oil penetration in the film and favours localisation close to the polar head. In this case, it is reasonable to suppose the formation of hydrogen bonds between the phenolic hydroxyl group of the compound and the protonated amine (${\mathrm{NH}}_{3}^{+}$) of the DMPE headgroup, which can favour the film compaction, in addition to the hydrophobic attraction between the SEO hydrocarbon ring and the hydrocarbon chains of the lipid. Evidence of this interaction was observed in the investigation of the surface potential of PE monolayers interacting with pure carvacrol [37], where the association between this zwitterionic lipid and carvacrol reduces the surface potential of the film.

The effect observed on CL is even smaller, likely due to its nature as a Gemini lipid with a rigid spacer, which acts as a constraint for film compaction. If only electrostatic repulsion between SEO and the polar head is considered, this finding might seem difficult to explain and would be expected to cause greater electrostatic repulsion (and, consequently, cause a larger disturbance). However, the hydrophobic portion plays a significant role due to the presence of two unsaturated bonds in each chain, which generate considerable hindrance and significant chain mobility. This results in a more disordered state, which may favour the accommodation of SEO in the film, thereby limiting its disturbance effect.

The monolayer of the zwitterionic DMPC shows a small packing disturbance, as evidenced by the increase in the molecular area, along with a slight variation in anisotropy. The presence of a P-N dipole in the choline polar head allows significant freedom of association between SEO and this lipid. The lipid can orient its P-N dipole vector to maximize interaction with SEO, finding an optimal accommodation that also promotes choline-SEO association through cation–π interaction [43]. A synergistic effect of the association between PC and thymol, which leads to the formation of domains in these bilayers, has been proposed as a key mechanism to explain thymol’s ability to penetrate PC films [23].

The analysis of the molecular area in monolayers gives an overall indication of packing modification and suggests that SEO exerts the largest effect in DMPG monolayers, followed by DMPE, CL, and DMPC. By comparing this finding with those obtained by DPH anisotropy on bilayers, it can be concluded that DMPG causes the largest variation in the deep region of the hydrocarbon chain. This localisation justifies the strong oil–lipid association, which may cause the detergent effect evidenced by the negative values of ΔP, obtained upon SEO insertion on the monolayers at 30 mN/m. Unlike the other lipids, DMPG exhibits an increase in the surface compression modulus K at low SEO concentrations, followed by an almost imperceptible decrease compared to pure film. At the same concentrations of SEO, the other lipids undergo a marked decrease in K. The peculiar effect observed in DMPG is generally attributed to an elastic response of the film, for an almost-liquid monolayer close to the lipid melting transition. In this condition, the higher flexibility of the film allows for an easier accommodation of SEO molecules. This behaviour significantly differs from the behaviours observed in the other more rigid lipid monolayers, such as DMPC and DMPE, which are not able to rearrange in response to the compression and, thus, undergo fluidization. Similar behaviours of the compression modulus were observed in negatively charged DPPS monolayers after thymol addition [24]. These results offer further support to the effect of modulation of the lipid–SEO interaction connected to the nature of the polar head.

The results shown in the present study strongly indicate the presence of the interaction between SEO and the investigated lipids monolayers, modulated by the details of the lipid headgroup. It is worth mentioning that while mammalian cell membranes are mainly composed of PC and PE lipids, bacterial membranes consist of varying ratios of PE, CL, and phosphatidylglycerol (PG). Gram-negative bacteria are characterized by membranes with a high content of PE, while Gram-positive bacteria, such as *S. aureus* and *S. pneumoniae*, contain only PG and CL (58% PG and 42% CL in S. aureus) [25]. The observed differences in the effect of SEO—depending on the polar head of the lipid—further support the potential use of this compound in pharmaceutical processes involving interactions with cell membranes and other bio-interfaces, such as biofilms [6,47], where the efficient transport of oil through the cell wall could facilitate the interaction with multiple molecular sites on the microbial cell membrane. Although the approach based on lipid Langmuir monolayers has intrinsic limitations, it remains a valuable tool for offering initial insights into the molecular mechanism of the interaction of this oil with the outermost layer of the cell.

The effects observed in this paper emerge from the examination of the thermodynamic properties of the system, connected to the supramolecular arrangement of SEO and lipids, which is not immediately evident by using the typical pharmaceutical approaches. For this reason, our results may encourage further studies on the complex drug–membrane systems, which take into account the nature of the drug and the physical–chemical characteristics of the membrane models employed, such as membrane organization, partitioning, film expansion, surface rheological properties, and preferential localization of the drug.

## 6. Conclusions

In this study, we carried out a proof-of-concept set of experiments using simplified models of cell membranes, known as lipid monolayers, to investigate their interaction with *Satureja Montana* essential oil (SEO). The commercial sample of SEO used, which was previously shown to exhibit significant biological activity, alters the thermodynamic properties of the membrane. In detail, SEO modifies lipid–lipid interactions, expands all the investigated monolayers, increases membrane fluidity and, consequently, changes the physicochemical properties of the film.

Our findings highlight the impact of the lipid polar head and the nature of aliphatic chains on the interaction between SEO and monolayers, emphasizing the significant role of electrostatic interactions. This investigation serves as an initial study on this sample. The results provide insight for exploring, at the molecular level, the interactions of potential natural drugs with more complex systems that more closely resemble cellular membranes.

In future works, we will aim to extend this approach to lipid mixtures to develop more realistic membrane models of bacteria. We hope that these findings can contribute to a deeper understanding of the interaction between SEO and cell membrane surfaces, providing valuable molecular insights into the pharmaceutical properties of this drug. 

## Figures and Tables

**Figure A1:**
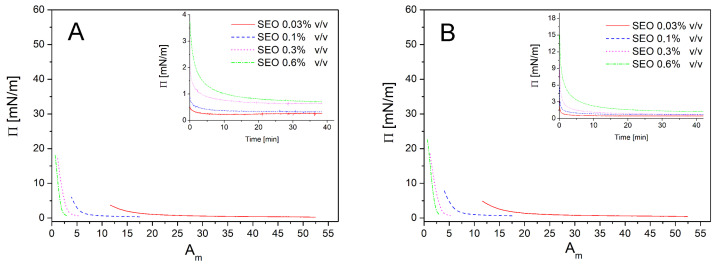
The surface pressure–area Π-$A_{m}$ isotherms of SEO monolayers, obtained by depositing an ethanolic solution of SEO at the air–water interface (**A**) or by injection under the interface (**B**), at different concentrations, as indicated in the label (continuous line: 0.03% *v*/*v*, dash: 0.1% *v*/*v*, dot: 0.3% *v*/*v*, dash–dot: 0.6% *v*/*v*), recorded after approximately 40 min after deposition to enable the stabilisation in surface pressure, as shown in the inset. Note the different scales for the vertical axis of the surface pressure of the inset.

**Figure A2:**
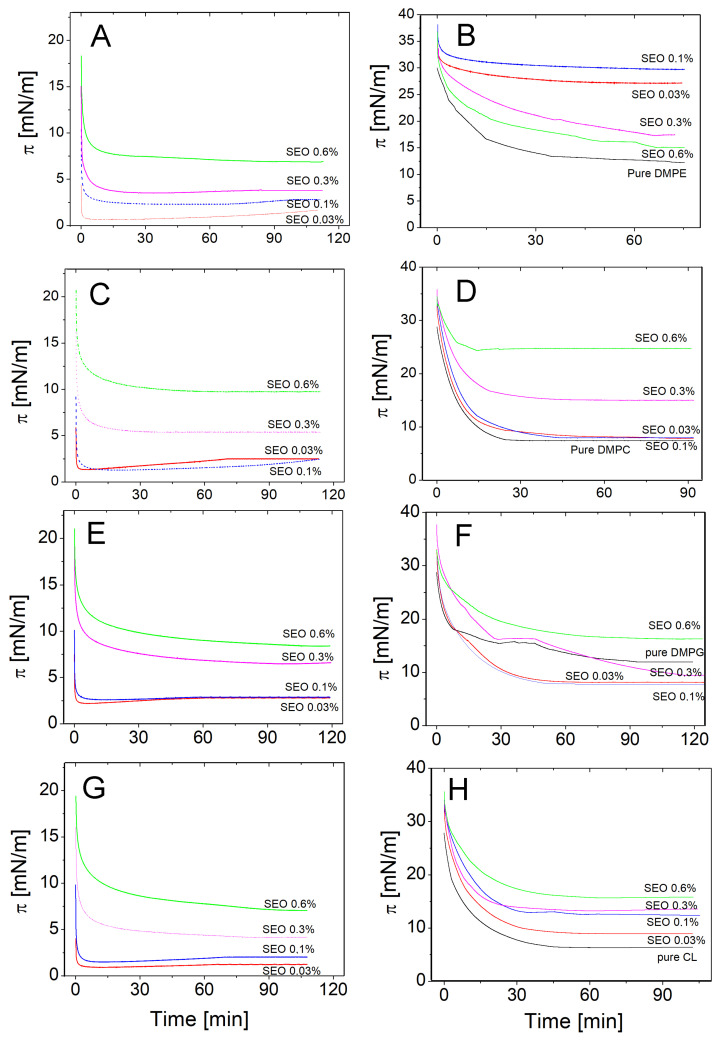
Relaxation behaviour of lipid monolayers after SEO injection at null surface pressure (**left** panels) and at 30 mN/m (**right** panels) for DMPE (**A**,**B**), DMPC (**C**,**D**), DMPG (**E**,**F**), and CL (**G**,**H**). Note the non-monotonous relaxation behaviour of DMPG upon injection of SEO at 30 mN/m (panel **F**), in the surface pressure range of 10–20 mN/m, where the LE-LC transition occurs.

## Data Availability

The original contributions presented in this study are included in the article/Appendix A. Further inquiries can be directed to the corresponding authors.

## Appendix A. Additional Data

### Appendix A.1. Surface Pressure Isotherm of SEO

To investigate the behaviour of SEO at the air–water interface, 50 μL of an ethanolic solution of SEO, diluted to various concentrations, was deposited on the HEPES subphase. The isotherm was recorded after stabilization of the surface pressure (see Figure A1A and the inset showing the variation in surface pressure post-deposition over time). Our findings indicate that SEO can form unstable monolayers, which reach surface pressure values lower than those of lipid monolayers. It is important to note that the isotherms obtained by depositing different concentrations of oil do not overlap as expected, thus demonstrating the significant loss of molecules, likely due to the volatility of SEO or molecular loss in the subphase. This is linked to the high solubility of ethanol in water, a phenomenon previously observed in other investigations on essential oils [30]. The same trend was observed when SEO was injected under the interface (see Figure A1B and the inset, showing the behaviour in surface pressure after injection). The comparison of the maximum surface pressure values reached by the film prepared by two different methods—deposition at the interface or injection under the subphase—shows that the increase in surface pressure is higher when SEO is injected under the subphase. This result can be explained by the different extents of evaporation occurring in the two protocols. When SEO is deposited at the interface, the molecules come in direct contact with the air, which favours evaporation. In contrast, with injection under the interface, the molecules may aggregate to form three-dimensional structures that can adsorb to the water surface, causing a larger increase in surface pressure.

### Appendix A.3. Liposome Size by Dynamic Light Scattering

The hydrodynamic diameter of lipid vesicles was measured using a dynamic light scattering (DLS) technique, employing a MALVERN Nano Zetasizer apparatus equipped with a 5 mW HeNe laser (Malvern Instruments LTD, UK). In DLS, the autocorrelation function of the scattered intensity is analysed as a function of decay times to obtain the distribution of diffusion, D, coefficients of the particles. This distribution is then converted into a distribution of apparent hydrodynamic diameters $2R_{H}$ using the Stokes–Einstein relationship, $2R_{H}=k_{B}T/3\pi \eta D$, where $k_{B}T$ is the thermal energy and η is the solvent viscosity.

As the preliminary analysis, the cumulant method was used to find the average hydrodynamic diameter, $2R_{H}$, and the polydispersity index (PDI) [48]. The intensity-weighted $2R_{H}$ obtained via cumulant analysis is the most direct and robust measure of the size, since it does not rely on the specifics of the scattering model. Moreover, it is derived from the initial portion of the autocorrelation function, where the signal-to-noise ratio is the highest. However, the size obtained by this method represents an average value over the entire sample and it may not be representative when the PDI values exceed ∼0.3, thus indicating that the size distribution might exhibit multiple peaks. To address this issue, an intensity-weighted NNLS (non-negative least squares) algorithm was applied to determine the size and size distribution of the different populations present in the dispersion [49]. In the intensity-weighted analysis, the scattered intensity is proportional to the sixth power of the particle size, which biases the resulting values towards a larger size [50]. If there are two or more distinct-sized populations, the NNLS number-weighted analysis was used to determine the relative percentages of the populations and identify the most abundant one.

The values of hydrodynamic diameter $2R_{H}$, determined by cumulant analysis, show that all the lipids can form vesicles with a mean size in the range of 100–200 nm and a relatively high PDI value. For all the samples, the NNLS analysis confirms that the vesicles in this size range represent the most abundant population. The addition of SEO reduces the values of PDI for all the lipids, thereby indicating that the suspension becomes more homogeneous.

An increase in size is observed for DMPG. This may be due to the higher affinity of SEO with the region of hydrophobic chains, which favours localization in this portion of the bilayer and decreases the vesicle’s curvature radius, thereby increasing its size. This result aligns with the indications from DPH anisotropy on lipid bilayers.

**Table A1:** Results of the DLS measurements on DMPC, DMPE, DMPG and CL liposomes, in the absence of SEO and after a 1 h incubation with SEO. Hydrodynamic diameter $2R_{H}$ and PDI are determined by the cumulant method; PK1 and % refer to the average value of the diameter and the relative abundance of the main component determined by the NNLS size distribution analysis.

Sample	$2R_{H}$± SD (nm)	PDI ± SD	PK1 (nm)—%
DMPC	231 ± 24	0.312 ±0.083	208–90%
DMPC + SEO	213 ± 11	0.17 ± 0.020	242–99%
DMPG	71 ± 19	0.59 ± 0.11	62–69%
DMPG + SEO	129 ± 10	0.211 ± 0.054	138–99%
DMPE	218 ± 16	0.496 ± 0.070	104–95%
DMPE + SEO	220 ± 4	0.245 ± 0.016	235–98%
Cardiolipin	350 ± 10	0.523 ± 0.011	70–90%
Cardiolipin + SEO	363 ± 11	0.311 ± 0.030	83–98%
